# Increased Mortality in Elderly Patients Admitted with Hyponatremia: A Prospective Cohort Study

**DOI:** 10.3390/jcm10143059

**Published:** 2021-07-10

**Authors:** Petros Ioannou, Symeon Panagiotakis, Emmanouela Tsagkaraki, Constantinos Tsioutis, Konstantinos Fragkiadakis, Achilleas Gikas, Theodosios D. Filippatos

**Affiliations:** 1 Department of Internal Medicine, University Hospital of Heraklion, 71110 Heraklion, Greece; symeon.panagiotakis@gmail.com (S.P.); emmanutsa17@gmail.com (E.T.); fragkiadakisk@hotmail.com (K.F.); gikas@med.uoc.gr (A.G.); 2 School of Medicine, European University Cyprus, Nicosia 2404, Cyprus; k.tsioutis@euc.ac.cy

**Keywords:** geriatric, hyponatremia, older, syndrome of inappropriate anti-diuresis, thiazides

## Abstract

Hyponatremia is the most common electrolyte disorder, commonly affecting older hospitalized individuals; however, the literature is not clear regarding its effect on mortality. The aim of this 2-year observational prospective cohort study was to evaluate the mortality and re-admission rates, the clinical and laboratory characteristics and the causes of hyponatremia in patients older than 65 years admitted with a corrected serum sodium of 130 mEq/L or less in an internal medicine ward of a tertiary Greek university hospital. During the observation period, 138 patients (mean age 80.5 years, 36.2% male) fulfilled the inclusion criteria and were prospectively followed for 1 year after admission. Symptoms of hyponatremia were present in 59.4% of patients. Hypovolemia was the main sole cause of hyponatremia, but in about one third of patients, hyponatremia was multifactorial. Only a low proportion of patients (12.3%) fulfilled the criteria of the syndrome of inappropriate antidiuresis (SIAD) at admission according to the current guidelines. The re-admission rates at 3- and 12-months following discharge was 34.2% and 51.8%, respectively. Mortality during hospitalization was 17.4% and was higher compared to non-hyponatremic admitted older patients, while the total mortality at 1 year after admission was 28.3%, indicating that hyponatremia at admission is a marker of significant mortality during and after hospitalization in elderly patients.

## 1. Introduction

Hyponatremia is the most common electrolyte disorder in both community and hospitalized patients and is associated with adverse outcomes and mortality, especially when it is moderate or severe (sodium levels less than 130 and 125 mEq/L, respectively) [1]. Its prevalence is even higher in older individuals due to physiologic changes that involve reduced total body water volume and lower urine concentrating ability [2]. Hyponatremia can manifest with a variety of symptoms, most commonly nausea, vomiting, dizziness, confusion, seizures and coma. Historically, the diagnostic evaluation of hyponatremia involves the assessment of the patient volume status, which can be quite subjective and unreliable, especially in older individuals [3]. Moreover, the work up of hyponatremia in older patients is also problematic due to more comorbidity, medications and other underlying conditions that could be associated with hyponatremia [4]. A common cause of hyponatremia with a problematic diagnosis in older individuals is the syndrome of inappropriate antidiuresis (SIAD), which is associated with water retention and dilutional hyponatremia due to excessive antidiuretic hormone (ADH) excretion. SIAD is reported to be more common in older individuals and its diagnosis may be problematic due to multifactorial etiology [5,6].

Hyponatremia is the most common electrolyte disorder but its reported frequency varies according to the healthcare setting and the definition of hyponatremia [7,8]. Additionally, data in older individuals is scarce in South European countries, especially in Greece. Moreover, there are conflicting data regarding the question if hyponatremia is associated with increased mortality in older patients (>65 years) [5,9,10,11,12]. The aim of this 2-year prospective cohort study was to evaluate re-admission and mortality rates during the following 12 months, the clinical and laboratory characteristics and the cause of moderate/profound hyponatremia in elderly patients admitted to a university hospital medical ward.

## 2. Materials and Methods 

This is a 2-year prospective cohort study of patients admitted to the Internal Medicine Department of the tertiary University Hospital of Heraklion, Crete, Greece. The study was approved by the ethics scientific committee of the hospital.

### 2.1. Inclusion and Exclusion Criteria

Inclusion criteria:

- Patients admitted to the Internal Medicine Department;
- Age 65 years or more;
- Moderate (serum sodium 126 mEq/L–130 mEq/L) or profound hyponatremia (serum sodium equal or less than 125 mEq/L). We have chosen the threshold of 130 mEq/L as it is associated with increased adverse outcomes and mortality in the general population and difficulties in correction [1,13].

Exclusion criteria:

- Non-hypotonic hyponatremia (hyponatremia associated with hyperglycemia, mannitol, glycine, radiocontrast media, maltose, hypertriglyceridemia, cholesterol, protein, immunoglobulins) [13];
- Serum sodium >130 mEq/L after correction for glucose according to the Hiller equation [14].

**Figure 1:**
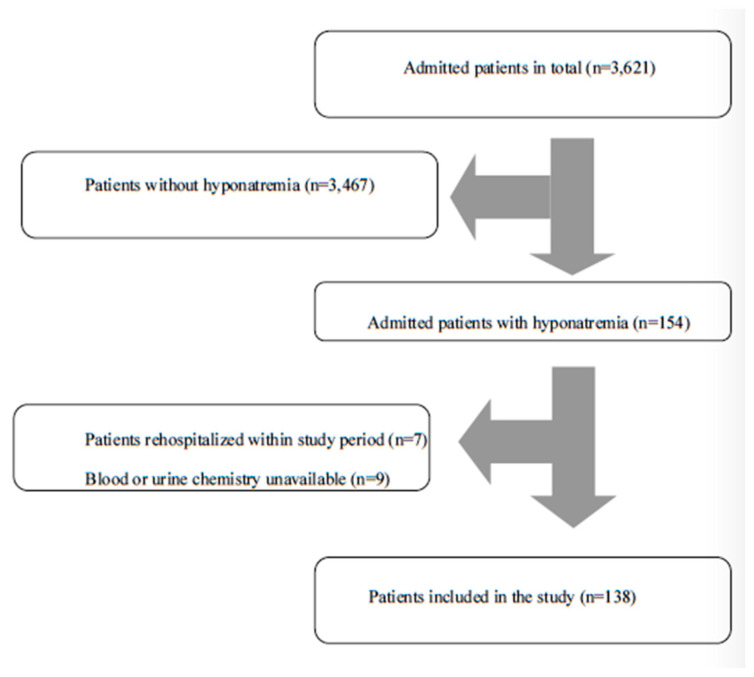
Patient selection flowchart.

The flowchart of patient inclusion is presented in Figure 1.

### 2.2. Demographics, Biochemical Analyses and Follow-Up

Data regarding demographics (age, gender), medical history, previous episodes of hyponatremia, clinical and laboratory parameters associated with hyponatremia (such as sodium, glucose, creatinine, urea, uric acid, thyroid-stimulating hormone (TSH), cortisol, proteins, cholesterol and triglycerides in serum and sodium, creatinine, urea and uric acid in urine) were collected. Presence of symptoms was assessed at admission and included all symptoms associated with hyponatremia (for example nausea, vomiting, dizziness and muscle cramps). In the case where symptoms could be either a cause or a consequence of hyponatremia (e.g., vomiting), a decision regarding whether the symptom was due to hyponatremia was made on clinical grounds based on the final diagnosis and the clinical response after resolution of hyponatremia. Functional assessment for activities of daily living (ADLs) was performed at the time of admission using the modified Barthel ADL index [15].

Patients were followed up to 12 months after admission and patient outcomes including mortality and re-admissions within 3 and 12 months were noted. If a patient had more than one admission, only the first admission was included in analyses.

### 2.3. Evaluation of Hyponatremia

The initial work up for causes of true hyponatremia was performed according to the established diagnostic algorithms. Because an osmometer was not readily available, we used urine special gravity < 1003 as a threshold for the diagnosis of maximally diluted urine, which is an indicator of water intoxication, beer potomania or low solute intake (tea and toast) diet. After that, the algorithm is based on urine biochemistry, followed by the patient’s volume for further identification of the cause of hyponatremia [13]. A patient was considered to be hypovolemic if they had clinical signs of orthostatic hypotension associated with a compatible history or indicative laboratory values such as urea to creatinine (in mg/dL) ratio greater than 57 [16]. The diagnosis of SIAD was initially based on previously characterized criteria [17], namely the presence of hypotonic hyponatremia, inappropriate urine concentration (urine special gravity > 1003 that corresponds to a urine osmolarity > 100 mOsm/kg), increased urine sodium (>30 mEq/L), clinical euvolemia and the absence of hypothyroidism, adrenal failure or kidney failure [17]. An estimated glomerular filtration rate (eGFR) < 30 mL/min/m^2^ determined according to the EPI-CKD (Chronic Kidney Disease Epidemiology Collaboration equation [18]) was used as a threshold for exclusion of SIAD and as a threshold for advanced kidney disease-associated hyponatremia. Hyponatremia in patients receiving thiazide diuretics was categorized as thiazide-associated hyponatremia.

### 2.4. Statistical Analyses

Data are presented as number (%) for categorical variables and median (interquartile range, IQR) or mean (+/− standard deviation, SD) for continuous variables. Categorical data were analyzed with Fisher’s exact test or the Pearson’s chi-square test, as appropriate. Continuous variables were compared using Student’s t-test for normally distributed variables and the Mann–Whitney U-test for non-normally distributed variables. All tests were two-tailed and *p*-values < 0.05 were considered to be significant. A univariate linear regression analysis was conducted to identify clinical, epidemiological and laboratory factors associated with mortality of hyponatremic patients during admission. All abovementioned statistics were calculated with GraphPad Prism 6.0 (GraphPad Software, Inc., San Diego, CA, USA). A multivariate logistic regression analysis was conducted to evaluate the effect of factors that were previously identified in the univariate analysis model as factors with a *p*-value lower than 0.05. Multivariate analysis was performed using SPSS version 23.0 (IBM Corp., Armonk, NY, USA).

## 3. Results

During the 2-year study period, 154 out of 2833 admissions (55.7% female, *n* = 1577) of patients older than 65 years old fulfilled the inclusion criteria. Seven of these cases were re-admissions and were excluded from the study, while in nine cases blood or urine chemistry at admission was unavailable, resulting in a final sample of 138 patients with hyponatremia (Figure 1). The mean age was 80.6 years and 36.2% of the patients were male. Interestingly, 37.7% of the sample had had hyponatremia at least once in the past. The most common underlying comorbidities were hypertension, chronic kidney disease, heart failure, diabetes mellitus, coronary artery disease and dementia. The median Barthel Index was 17 (IQR 8–20) (Table 1). Of note, a chi-square test revealed a statistically significant difference between gender and presence of hyponatremia (*p* < 0.0001), confirming that older patients with hyponatremia were more likely to be female.

Symptoms of hyponatremia were present in 59.4% of patients for an average of 7.7 days before presentation. Among symptomatic patients, the most common symptoms were dizziness (58.5%), nausea (56.1%), vomiting (42.7%) and headache (13.4%). Notably, 10.1% of patients were confused at admission and 9.4% had had a fall during the last 14 days before presentation.

Hypovolemia was the main sole cause of hyponatremia (28.3%). Thiazide use was detected as the sole cause of hyponatremia in 9.4% (13 patients) and as a main cause of hyponatremia in 21.1% (38 patients) of the sample. In 31.9% of all patients (44 patients), multifactorial hyponatremia (at least two causes of hyponatremia) was found (Table 2).

**Figure 2:**
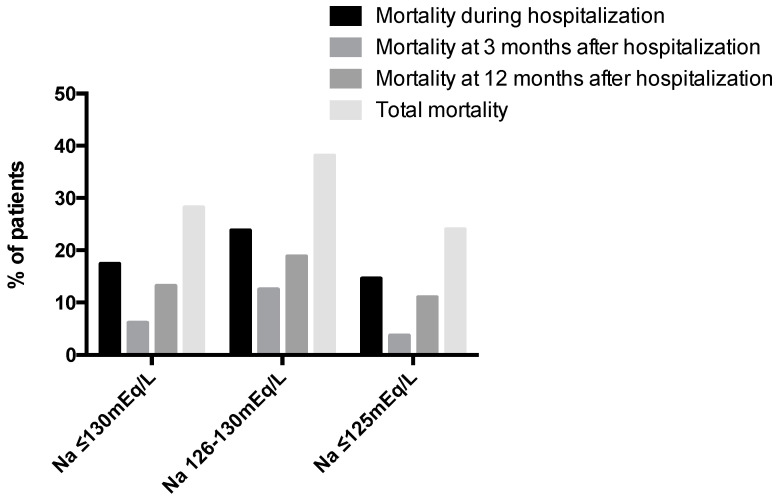
Mortality of patients in regards to the sodium level.

The median length of stay was 6 days (IQR 4–9 days). The mortality rate during hospitalization was 17.4% (24 patients out of 138 patients), which was significantly higher than the mortality rate observed in hospitalized patients without hyponatremia (13.4%; 316 patients out of 2679 patients > 65 years old) during the same period of observation (*p* = 0.049). In our cohort, the re-admission rate at 3- and 12-months following discharge was 34.2% and 51.8% (39 and 59 out of 114 patients, respectively). Among the 114 patients who were initially discharged, 28.9% had at least one episode of hyponatremia within the next 12 months. The mortality rate at 3- and 12-months following discharge was 6.1% and 13.2% (7 and 15 out of 114 patients, respectively), whereas the total mortality at the end of the 12-month follow-up period in the whole sample of hyponatremic patients was 28.3% (39 out of 138 patients). Serum sodium levels at admission lower than or equal to 125 mEq/L were not associated with increased length of stay, re-admission or mortality rates compared with sodium levels 126–130 mEq/L (Figure 2).

Patients with SIAD consisted of only 12.3% (17 patients) of our cohort of patients with hyponatremia. Their main clinical and biochemical characteristics are shown in Table 3. Interestingly, patients with SIAD had a higher 12-month re-admission rate compared to other euvolemic patients with hyponatremia. If instead of using an eGFR lower than 30 mL/min/m^2^ as an exclusion criterion for SIAD, the threshold of 60 mL/min/m^2^ was used, four patients would have been excluded from the SIAD group.

Patients with thiazide-associated hyponatremia (as a sole or main cause of hyponatremia) were more likely to have a higher Barthel index and to be symptomatic. Furthermore, they had lower corrected serum sodium and they also had lower mortality, lower re-admission rate at 12 months, lower mortality at 12 months and lower incidence of future hyponatremia.

Regression analysis was performed in order to identify factors independently associated with mortality in hyponatremic patients. Initially, a univariate linear regression analysis of age, gender, duration of hospitalization, presence of symptoms of hyponatremia, thiazide use, Barthel Index, diagnosis of SIAD during this hospitalization, laboratory parameters in serum such as corrected sodium, urea, uric acid, proteins, creatinine, TSH, chloride, osmolarity and laboratory parameters in urine such as urea, uric acid and sodium with mortality during admission was carried out. Then, we performed a multivariate logistic regression analysis with factors identified in the univariate analysis (symptomatic hyponatremia, thiazide use, Barthel Index, serum uric acid, proteins and TSH) to be associated with mortality. The analysis identified thiazide use and increased Barthel Index to be independently negatively associated with mortality during admission and increased serum uric acid to be independently positively associated with mortality during admission. The results of the regression analysis are shown in Table 4.

## 4. Discussion

To the best of our knowledge, this is the first prospective study specifically aimed at providing data on hyponatremic elderly patients regarding re-admission and mortality rates at 1 year follow-up after admission in a medical ward. Additionally, to the best of our knowledge, this is the first study in Southern Europe describing the characteristics and outcomes of hyponatremia in elderly admitted patients.

Mortality during admission is rather variable in previous reports of hospitalized patients with hyponatremia, ranging between 5.9% and 27.0%, possibly due to different sample characteristics [5,19,20,21]. In our sample of elderly patients, the mortality rate during hospitalization was 17.4%, which was significantly higher than in patients that did not have hyponatremia. Thus, our results are consistent with the findings in the general hospitalized population showing hyponatremia to be associated with higher in-hospital mortality, as well as higher mortality after one and five years [22]. Similarly, a prospective observational German study with paired controls of hospitalized older patients showed that hyponatremia (Na^+^ < 130 mEq/L) is associated with a higher likelihood of delirium and an elevated in-hospital mortality (10.6% versus 2.1%; *p* = 0.005) [23]. Interestingly, a multivariate logistic regression analysis identified thiazide use and increased Barthel Index to be independently negatively associated with mortality. Indeed, thiazide-associated hyponatremia, as discussed below, seems to be associated with a milder hospital course. The identification of association of increased Barthel Index with less mortality is not a new finding, since it is known in the literature that Barthel Index at hospital admission is associated with mortality in geriatric patients [24]. The identification of uric acid as an independent factor associated with mortality in older patients with hyponatremia is compatible with the findings of several studies that show a correlation between uric acid and mortality, but mostly in patients with cardiovascular diseases, diabetes and chronic kidney disease [25,26,27,28].

In line with other studies, almost 1 out of 3 patients in our cohort had a previous history of hyponatremia [29]. Furthermore, almost 1 out of 4 patients had a future event of hyponatremia within 12 months after discharge. Re-admission and mortality rates in the following months after discharge were high, indicating a high risk for these outcomes in elderly patients admitted with hyponatremia. It should be mentioned that data regarding whether mortality increases in parallel with the severity of hyponatremia are conflicting [19,30,31,32,33,34]. In our study, the length of hospital stays as well as the re-admission and mortality rates were not increased in patients with severe hyponatremia compared to patients with moderate hyponatremia, indicating that corrected sodium levels do not represent an independent factor but a marker of increased morbidity/mortality in geriatric patients.

In our cohort, more hyponatremic patients were women (a proportion quite higher than that in the general population, out of 2833 patients older than 65 years that were admitted in the same period); this is in line with other studies in the general population, even though evidence suggests that the female gender as a risk factor may be confounded by low body weight [5,19,29]. Our sample had more comorbidities compared to the literature [19,31], as was anticipated since we only included older patients. A very high proportion of patients in our study were using angiotensin-converting enzyme (ACE) inhibitors, angiotensin receptor blockers, thiazide diuretics and furosemide, which is in line with other studies in the literature, which show that these drugs predispose patients to hyponatremia [35,36]; however, for some drugs, such as furosemide, this association may not reflect a causal role with hyponatremia but a causal role between the indication for which the medication is prescribed (more typically heart failure) and hyponatremia.

Symptoms of hyponatremia in our study were similar to those in the literature [29]. An important observation is that nearly 1 out of our 10 patients had had a fall the last 14 days before presentation, which is in line with the literature and raises special concerns given the fact that hyponatremia is associated with an increased risk of bone fracture in patients older than 60 years old due to bone demineralization and increased risk of falls [37,38].

In our study, which included elderly patients with moderate/profound hyponatremia, hypovolemia was the most common sole etiologic factor. This is not the case in younger individuals [39]. Although evaluation of hyponatremia according to most diagnostic algorithms mainly relies on the evaluation of the patient’s volume [3], this can be unreliable in older patients [40]; moreover, there is no reliable biomarker of intravascular volume and hydration [41,42]. Additionally, hyponatremia is frequently thought to have a multifactorial etiology, especially in older individuals [5,43]. In our study, about 1 out of 3 patients had hyponatremia of multifactorial etiology. The increased prevalence of heart and kidney failure in the elderly seems to play a role in the multifactorial etiology of hyponatremia in this population. The high percentage of multifactorial etiology makes the applicability of diagnostic algorithms for the evaluation of hyponatremia more difficult in elderly individuals.

Diagnosing SIAD may sometimes be difficult [44]. The rate of SIAD was rather low (12.3%) in our sample, an observation that can be attributed to the rather strict criteria of this diagnosis [13,17]. According to Spasovski et al. [13], diagnosis of SIAD is a diagnosis of exclusion and requires, among others, the presence of clinical euvolemia, urine sodium concentration > 30 mEql/L with normal dietary salt and water intake, absence of adrenal, thyroid, pituitary or renal insufficiency and absence of recent diuretic use. It is rather difficult for an older person to fulfil all of these criteria. It is of note that in the guidelines published by Spasovski et al., there is no cutoff point for eGFR or creatinine [13]. This is of utmost importance, since eGFR declines with aging, implying that a significant proportion of patients with a reduced eGFR but without significant tubulointerstitial or glomerular damage that would pathophysiologically justify exclusion of SIAD diagnosis, may be precluded from this diagnosis. For example, in our cohort, 63.8% of patients had an eGFR lower than 60 mL/min/1.73 m^2^, that could, theoretically, have been used as a cutoff point instead of 30 mL/min/1.73 m^2^ that was used, since, according to the KDIGO and KDOQI guidelines, persistence of eGFR below 60 mL/min/1.73 m^2^ for at least three months, irrespective of the cause, is sufficient to diagnose chronic kidney disease [45,46]. In this study, we chose to use a cutoff point of 30 mL/min/1.73 m^2^, since we believe that it more precisely reflects advanced kidney disease, in which the underlying subclinical tubulointerstitial damage that affects urine-concentrating ability would indeed be present. If an eGFR lower than 60 mL/min/1.73 m^2^ had been used, the rate of SIAD would have been lower, with only 13 patients (9.4%) diagnosed with SIAD.

Thiazide-associated hyponatremia seems to have some specific characteristics that make it quite different from the other causes of hyponatremia in elderly patients. Patients with thiazide-associated hyponatremia are more frequently women, more likely to be symptomatic, have lower serum sodium and have a better clinical course with a lower mortality and a lower recurrence rate. Furthermore, thiazide use was also found with the regression analysis to be independently associated with reduced mortality. To our knowledge, there has been no study until now directly comparing the characteristics of the patients with and without thiazide-associated hyponatremia in geriatric patients, even though there are studies describing the characteristics of patients with thiazide-associated hyponatremia both in the general population and in older individuals [6,47,48].

Our study has some limitations that we should acknowledge. First of all, this study included only patients hospitalized in the Internal Medicine Department, thus, patients not admitted, as well as patients admitted in other wards may have been missed. However, it is rather uncommon for patients with moderate or profound hyponatremia to be discharged and most medical patients with such low sodium values are admitted in our department. Furthermore, a few patients with hyponatremia that did not have urine chemistry available were excluded from the analysis. In addition, despite the fact that our hospital is a referral center for Southern Greece, a few post-discharge visits to other healthcare centers may have been missed and therefore the re-admission rate may have been underestimated. Moreover, assessment of hyponatremia relies on the estimation of fluid volume and this can be problematic, since differentiation of hypovolemia from euvolemia can be difficult. Thus, any mistakes during the estimation of the patients’ fluid status may have affected the results of the current study. Additionally, it should be noted that choosing to exclude patients with an eGFR lower than 30 mL/min/1.73 m^2^ from the diagnosis of SIAD may seem arbitrary, however, the guidelines published by Spasovski et al. do not define a cutoff for the eGFR. Moreover, patients with mild hyponatremia, or patients with hyponatremia occurring in the hospital, were not included in this study. Finally, we do not have data for morbidity/mortality extending after the 12-month period after admission, thus the possible longer-term consequences of hyponatremia cannot be assessed, while, on the other hand, the causes of mortality were not reported.

## 5. Conclusions

To conclude, a significant proportion of older patients (approximately 25%) with hyponatremia were re-admitted or died during hospitalization or in the following 12 months after admission. Thus, hyponatremia on admission is associated with increased morbidity/mortality during the hospital stay and in the next year in elderly individuals. This study also underlines the difficulty of assessing geriatric patients who exhibit multifactorial hyponatremia in a significant portion.

## Figures and Tables

**Table 1:** Characteristics of admitted patients with moderate/severe hyponatremia.

Patient Characteristics	Na^+^ ≤ 130 mEq/L (*n* = 138)
Mean age, years (+/− SD)	80.6 (7.5)
Male (*n*, %)	50 (36.2)
Medical history	
Hypertension (*n*, %)	121 (87.7)
Chronic kidney disease (*n*, %)	88 (63.8)
Estimated GFR (EPI-CKD), mL/min/1.73 m^2^ (median, IQR)	50 (33.8–69)
Heart failure (*n*, %)	64 (46.4)
Diabetes mellitus (*n*, %)	50 (36.2)
Ischemic heart disease (*n*, %)	31 (22.5)
Dementia (*n*, %)	27 (19.6)
Chronic lung disease (*n*, %)	22 (15.9)
Cerebrovascular disease (*n*, %)	19 (13.8)
Median Barthel Index (IQR)	17 (8–20)
Hyponatremia in the past (*n*, %)	52 (37.7)
Chronic medications	
ACE inhibitors and angiotensin receptor blockers (*n*, %)	92 (66.7)
Thiazide diuretics (*n*, %)	51 (37)
Furosemide (*n*, %)	48 (34.8)
Calcium channel blockers (*n*, %)	48 (34.8)
Selective serotonin receptor inhibitors (*n*, %)	34 (24.6)
Acetylsalicylic acid (*n*, %)	29 (21)
Aldosterone receptor antagonists (*n*, %)	23 (16.7)
Antipsychotics (*n*, %)	16 (11.6)
Symptomatic (*n*, %)	80 (59.4)

ACE: angiotensin-converting enzyme; EPI-CKD: Chronic Kidney Disease Epidemiology Collaboration; GFR: glomerular filtration rate; IQR: interquartile range; SD: standard deviation.

**Table 2:** Causes of hyponatremia.

Hypovolemic Hyponatremia (*n* = 73, 52.9%)	Euvolemic Hyponatremia (*n* = 37, 26.8%)	Hypervolemic Hyponatremia (*n* = 28, 20.3%)
Overt fluid losses (*n* = 73, 52.9%)	Thiazide use (*n* = 18, 13%)	Decompensated heart failure (*n* = 24, 17.4%)
Infection (*n* = 37, 26.8%)	SIAD (*n* = 17, 12.3%)	Kidney failure (*n* = 3, 2.2%)
Thiazide use (*n* = 30, 21.7%)	Excessive water intake (*n* = 1, 0.7%)	Liver cirrhosis (*n* = 1, 0.7%)
At least two causes (*n* = 34, 24,6%)	Corticoid insufficiency (*n* = 1, 0.7%)	At least two causes (additional causes: thiazide use *n* = 3; decompensated heart failure *n* = 1) (*n* = 3, 2.2%)
	At least two causes (additional causes: urinary retention *n* = 3; hypothyroidism *n* = 3; kidney failure *n* = 2) (*n* = 7, 5.1%)	

**Table 3:** Comparison of the characteristics of euvolemic patients with and without SIAD.

Patient Characteristics	Euvolemic, SIAD (*n* = 17)	Euvolemic, No SIAD (*n* = 20)	*p*
Mean age (+/− SD)	77.7 (7.9)	77.8 (7.8)	0.9519
Male (*n*, %)	7 (41.2)	7 (35)	0.7447
Median Barthel Index (IQR)	16.5 (0–20)	19 (15.8–20)	0.1015
Hyponatremia in the past (*n*, %)	8 (57.1)	9 (45)	1
Symptomatic (*n*, %)	9 (52.9)	17 (85)	0.0689
Mean corrected serum sodium mEq/L (+/− SD)	121.6 (6.5)	117.5 (6.5)	0.0618
Serum uric acid md/dL (+/− SD)	3.1 (1.7)	3.6 (2)	0.4468
Mean urine sodium mEq/L (+/− SD)	71.4 (25.9)	63.2 (40.1)	0.4752
Mean fractional excretion of sodium % (+/− SD)	1.5 (1.7)	1.3 (0.9)	0.6314
Mean plasma osmolarity mOsm/kg (+/− SD)	254.4 (14.1)	249.3 (12)	0.2423
Mean fractional excretion of uric acid % (+/− SD)	24.5 (23.8)	19.5 (18.3)	0.4881
Median length of hospitalization in days (IQR)	6 (4–8.5)	5 (4–7)	0.4973
Overall mortality (*n*, %)	3 (17.6)	0 (0)	0.0875
Re-admission at 3 months among survivors (*n*, %)	5 (29.4)	1 (5)	0.0752
Re-admission at 12 months among survivors (*n*, %)	10 (58.8)	3 (15)	0.0075
Hyponatremia after the hospitalization among survivors (*n*, %)	8 (57.1)	3 (15)	0.0689
Mortality at 3 months among discharged patients (*n*, %)	2 (14.3)	0 (0)	0.1622
Mortality at 12 months among discharged patients (*n*, %)	2 (14.3)	0 (0)	0.1622

SIAD: Syndrome of inappropriate anti-diuresis; SD: standard deviation; IQR: interquartile range.

**Table 4:** Results of the regression analysis of mortality in patients with hyponatremia.

	Univariate Analysis *p*	Multivariate Analysis *p*	OR (95% CI)
Symptomatic hyponatremia	0.0457	0.988	0.992 (0.335–2.936)
Thiazide use	0.0012	0.041	0.176 (0.033–0.934)
Barthel Index (per unit)	<0.0001	0.005	0.904 (0.843–0.971)
Uric acid (per mg/dL)	0.0032	0.001	1.278 (1.099–1.485)
Proteins (per g/dL)	0.0046	0.339	0.892 (0.706–1.127)
TSH (per mU/L)	0.0073	0.617	0.998 (0.99–1.006)

CI: confidence intervals; OR: odds ratio.

## Data Availability

The data presented in this study are available on request from the corresponding authors.
